# Plasma boron concentrations and risk of all-cause mortality in the general population

**DOI:** 10.1007/s00394-023-03319-1

**Published:** 2024-01-20

**Authors:** Paula Stürmer, Katharina Susanne Weber, Eike Andreas Strathmann, Wolfgang Lieb

**Affiliations:** Institute of Epidemiology, Kiel University, Niemannsweg 11, 24105 Kiel, Germany

**Keywords:** All-cause mortality, General population, Plasma boron, Cox regression

## Abstract

**Purpose:**

Higher dietary intake or higher circulating levels of the trace element boron have been associated with beneficial effects on human health. However, the relationship between plasma boron levels and survival in the general population is not known. Therefore, we aimed to assess the association between plasma boron concentrations and all-cause mortality in a population-based cohort from northern Germany (*n* = 863 individuals; median age 62.3 years, 42.8% women).

**Methods:**

Plasma boron concentrations (median 31.9 µg/L [22.9; 43.5]) were measured by inductively coupled plasma-mass spectrometry. Cox proportional hazards regression models adjusted for relevant confounders were used to associate plasma boron concentrations with all-cause mortality.

**Results:**

After a median follow-up time of 11 years, *n* = 99 individuals had died. In the overall sample, plasma boron concentrations were associated with all-cause mortality in the crude model (HR: 1.07 [95% CI 1.03–1.11] per 5-unit-increment). However, multivariable adjustment rendered the association non-significant (HR: 1.03 [95% CI 0.99–1.07]). Sex-stratified analyses revealed slightly higher mortality hazards with increasing plasma boron concentrations in women (HR: 1.11 [95% CI 1.03–1.18], *p*_Interaction_ = 0.034), but not in men (HR: 1.00 [95% CI 0.95–1.06]).

**Conclusion:**

We conclude that in a moderate-sized sample from the general population, higher plasma boron concentrations were associated with a higher risk of all-cause mortality in women, but not in men. Due to the low number of events in the female subsample (*n* = 27), this observation has to be interpreted with caution.

## Introduction

Boron is a chemical element that naturally occurs in rock, soil, and water. As a micronutrient, plant foods and drinking water are the main dietary sources of boron [1]. Experimental studies found boron to be paramount for various biological and physiological processes in plants, animals, and humans. Among others, in humans, beneficial physiological effects of boron on health outcomes were found in the context of bone health, immune function, and functions of steroid hormones [2].

We have previously reported associations of higher plasma boron concentrations with a lower body mass index (BMI) and lower C-reactive protein levels as well as with an overall more healthy diet in a community-based sample (similar to the one used in the present analyses) [3]. Moreover, in a sample from northern France, higher levels of boron in drinking water were not associated with adverse health effects [4]. While the majority of studies reported beneficial effects of boron supplementation [2], detrimental effects on reproductivity have been observed in rodents after excessive boron application [5].

Data on the association of boron with mortality are scarce. In a cohort of kidney transplant recipients, a higher urinary boron excretion was associated with longer survival [6]. However, to our knowledge, the association of plasma boron concentrations with mortality in the general population has not yet been reported. Therefore, we assessed the association of plasma boron concentrations with all-cause mortality in a moderate-sized population-based sample (*n* = 863) from the North of Germany.

## Methods

### Study sample

We conducted the present analyses in a subsample of the so-called “control cohort” of the popgen Biobank in Kiel, Germany [7]. Popgen was initiated in 2003 and is one of the largest academic biorepositories in northern Germany. Its initial focus was set on collecting DNA samples for genetic case–control studies with the “control cohort” serving as a reference sample. For this “control cohort”, between 2005 and 2007, 1316 individuals were recruited through population registries and from blood donors of the University Hospital in Kiel. Thereafter, the initial reference sample was transformed into a cohort and subjects were re-invited for a second examination cycle. Data collected during this second examination cycle (2010–2012) served as the baseline examination for our analyses relating circulating plasma boron to mortality. The assessment included a physical examination by trained personnel, as well as the answering of standardized questionnaires on demographics and lifestyle factors. Furthermore, different biosamples were collected, including EDTA plasma, serum, urine, and stool samples, and stored at − 80 °C until further analysis.

Plasma boron concentrations were measured via an inductively coupled plasma-mass spectrometry ICAP Q instrument (Thermo Fisher Scientific, Waltham, MA, USA) at Synalb (Jena, Germany) in accordance with DIN EN ISO 17294:2: 2017-01 with an uncertainty of measurement of 5%. Rhodium was used as an internal standard. Boron concentrations were available for *n* = 929 individuals. In 2022 (last date of follow-up: 19.07.2022), vital status of participants was ascertained by use of an online service from the central registration database. We excluded participants with missing data on sex, education, smoking status, and dietary intake, as well as those lost to follow-up. The final analytical sample comprised *n* = 863 individuals. The study was approved by the ethical review board of the Medical Faculty of the Kiel University (project identification code A 156/03; P2N reference number 2023-023). All participants gave written informed consent.

### Statistical analyses

For two-sided tests, statistical significance was considered at *p* < 0.05. We performed all analyses with SAS Enterprise Guide 7.1 (SAS Institute, Cary, NC, USA).

#### Association of plasma boron concentrations with all-cause mortality

According to plasma boron concentrations, participants were categorized into tertiles prior to analyses. The bottom tertile was set as reference for survival analyses with all-cause mortality as the endpoint. The underlying time variable was years alive between baseline and death or censoring in 2022. When modeled on a continuous scale, effect estimates were provided per 5-unit-increment of plasma boron concentrations. Kaplan–Meier curves were used to graphically display unadjusted associations between tertiles of plasma boron concentrations and all-cause mortality. Cox proportional hazards regression models were used to quantify associations. Model 1 was unadjusted, while model 2 was adjusted for sex and age. Model 3 further included BMI (kg/m^2^), Waist-to-Hip Ratio, years of education (≤ 9, 10, ≥ 11 years), smoking status (never [≤ 3 months duration], former [≥ 3 months duration], and current smokers), and physical activity (MET-h/week). The Schoenfeld residual method as well as inclusion of time interaction terms in the model were used to verify the proportional hazards assumption. As age did not meet this assumption, a time interaction term (survival time * age) was included in the Cox regression models which were adjusted for age as a confounder.

#### Sex-stratified and sensitivity analyses

In addition to analyses in the whole study sample, we performed sex-stratified analyses in the fully adjusted model. We tested for effect modification by sex by including a multiplicate interaction term (plasma boron concentrations * sex) in the model. To minimize the possibility of reverse causality, we performed a sensitivity analysis excluding individuals who died within 1 year after baseline.

#### Restricted cubic spline regression

We tested for nonlinear associations between plasma boron concentrations and all-cause mortality by use of restricted cubic splines analyses with knots placed at the 5th, 50th, and 90th percentile. The same covariates as in the Cox regression model 3 were applied and analyses were performed in the overall samples as well as stratified by sex.

#### Exploratory dietary pattern derivation by reduced rank regression

To elucidate if an association of plasma boron concentrations with all-cause mortality was due to plasma levels of this trace element or rather due to dietary intake influencing circulating levels of boron, we extracted an exploratory dietary pattern by use of a reduced rank regression (RRR) [8]. This method was already applied by our work group in a previous study based on the same original study sample [3]. However, as the sample sizes between both studies differ slightly, here, we derived a new RRR pattern to adequately reflect the current analytical sample. RRR analysis was performed using ln-transformed and sex-standardized values of 42 predefined food groups as predictor variables and ln-transformed plasma boron concentrations as the response variable. Food groups with factor loadings ≥ |0.2| were used to calculate a dietary pattern by adding up the intake of the respective food groups. In survival and stratified analyses as described above, the dietary pattern was included as a 1-standard deviation (SD) increment in the fully adjusted Cox regression model to associate it with all-cause mortality risk. Here, we additionally included daily total energy intake (kcal/day) as a covariate in the Cox regression model.

## Results

### Characterization of the study sample

Baseline characteristics of our elderly study sample (*n* = 863), stratified by sex and by survival status at the end of the follow-up period, are provided in Table 1. Median plasma boron concentrations were 31.2 µg/L [22.8; 44.4] in men and 33.5 µg/L [23.2; 44.9] in women. Over a median follow-up time of 11 years, *n* = 99 participants died, of which *n* = 27 were female. Median plasma boron concentrations were slightly higher in men (*p* = 0.0183) that died and markedly higher in women (*p* = 0.0007) that died than in individuals still alive at the end of the follow-up period.

**Table 1:** Characterisation of the overall study sample and stratified by mortality status and sex

	Overall sample (*n* = 863)		Deceased^a^ (*n* = 99)		Alive^a^ (*n* = 764)	
	Men (*n* = 494)	Women (*n* = 369)	Men (*n* = 72)	Women (*n* = 27)	Men (*n*= 422)	Women (*n* = 342)
Plasma boron concentrations in µg/L	31.2 [22.8; 42.4]	33.5 [23.2; 44.9]	35.0 [23.7; 50.5]	46.8 [32.1; 56.1]	30.7 [22.8; 41.4]	32.1 [22.5; 44.0]
Age in years	62.2 [55.0; 70.7]	62.6 [54.2; 71.3]	72.1 [63.7; 76.2]	71.1 [64.7; 76.4]	61.0 [53.7; 69.0]	62.1 [52.8; 71.1]
Survival time in years^a^	11.0 [10.0; 11.0]	11.0 [10.0; 12.0]	7.5 [5.0; 10.0]	8.0 [4.0; 10.0]	11.0 [10.0; 11.0]	11.0 [10.0; 12.0]
Height in cm	178.0 [172.5; 182.5]	163.5 [159.0; 168.5]	173.8 [167.8; 178.5]	159.5 [155.0; 167.0]	178.0 [173.0; 183.0]	163.5 [159.5; 169.0]
Weight in kg	86.0 [77.6; 95.1]	70.0 [61.8; 79.1]	83.9 [75.3; 90.2]	64.4 [60.4; 72.0]	86.4 [78.4; 95.7]	70.5 [61.8; 79.5]
BMI in kg/m^2^	27.0 [25.1; 29.7]	26.1 [23.0; 29.7]	27.3 [25.2; 30.4]	25.3 [24.0; 30.0]	27.0 [25.1; 29.5]	26.2 [22.8; 29.7]
WHR in cm	0.99 [0.90; 1.00]	0.88 [0.83; 0.92]	1.00 [0.97; 1.05]	0.88 [0.85; 0.92]	0.99 [0.94; 1.02]	0.88 [0.83; 0.92]
Diabetes^b^, *n* (%)	59 (12.0%)	24 (7.0%)	20 (28.2%)	2 (7.4%)	39 (9.0%)	22 (6.4%)
Hypertension^b^, *n* (%)	338 (68.4%)	216 (59.0%)	58 (80.6%)	21 (77.8%)	280 (66.0%)	195 (57.2%)
Physical activity in MET-h/week	82.8 [54.3; 125.3]	96.8 [65.5; 138.6]	72.3 [51.8; 123.5]	81.8 [53.0; 144.3]	83.6 [54.8; 125.3]	97.0 [66.3; 138.0]
Energy intake in kcal/day	2404.2 [2016.6; 2910.1]	1829.4 [1596.5; 2151.4]	2388.1 [1994.2; 3230.5]	1650.5 [1441.6; 2206.1]	2410.2 [2026.3; 2888.0]	1838.3 [1609.5; 2151.4]
Education						
≤ 9 years, *n* (%)	171 (34.6%)	129 (35.0%)	33 (45.8%)	14 (51.9%)	138 (32.7%)	115 (33.6%)
10 years, *n* (%)	138 (27.9%)	152 (41.0%)	17 (23.6%)	9 (33.3%)	121 (28.7%)	143 (41.8%)
≥ 11 years, *n* (%)	185 (37.5%)	88 (24.0%)	22 (30.6%)	4 (14.8%)	163 (38.6%)	84 (24.6%)
Smoking status						
Never, *n* (%)	166 (33.6%)	201 (54.0%)	16 (22.2%)	12 (44.4%)	150 (36.0%)	189 (55.3%)
Current, *n* (%)	70 (14.2%)	51 (14.0%)	10 (13.9%)	3 (11.2%)	60 (14.0%)	48 (14.0%)
Former, *n* (%)	258 (52.2%)	117 (32.0%)	46 (63.9%)	12 (44.4%)	212 (50.0%)	105 (30.7%)

Values of categorical variables are *n* (%) and of continuous variables are median [IQR]. All values except for survival time and mortality status are baseline characteristics collected at the second examination cycle of the popgen “control cohort”

*BMI* body mass index, *cm* centimeters, *kcal* kilocalories, *kg* kilograms, *m* meters, *MET-h* metabolic equivalent of task in hours, *WHR* waist-to-hip ratio

^a^At mortality follow-up in 2022

^b^Because of missing values in one subject, here total sample size *n* = 862

### Association of plasma boron concentrations with all-cause mortality

**Fig. 1:**
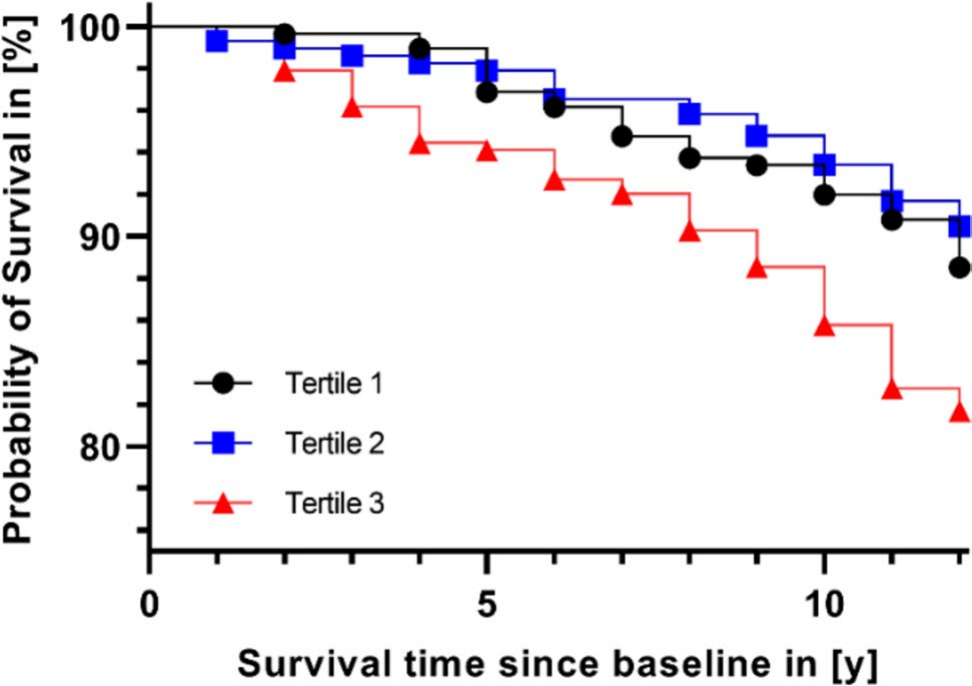
Unadjusted association between tertiles of plasma boron concentrations and all-cause mortality by use of Kaplan–Meier curves. By use of Log-Rank test, a significant association between tertiles of plasma boron concentrations and all-cause mortality was found (*p* = 0.0021). Artwork was created using GraphPad Prism version 9.4.1, GraphPad Software.

Figure 1 graphically displays the statistically significant association between tertiles of plasma boron concentrations and risk of all-cause mortality (*p*_Log-Rank Test_ = 0.0021). Individuals in the third tertile showed an 85% ([95% CI 1.15–2.97]) higher risk of all-cause mortality than participants in the lowest tertile in the unadjusted Cox regression model (Table 2). Likewise, when modeled as a 5-unit-increment of plasma boron, boron concentrations were associated with all-cause mortality risk (HR: 1.07 [95% CI 1.03–1.11]). However, associations did not hold true in the multivariable adjusted Cox regression models. Results of the sensitivity analysis, where participants who died within 1 year after baseline were excluded, did not differ from results obtained in the whole study sample (data not shown).

**Table 2:** Association of plasma boron concentrations with all-cause mortality using Cox proportional hazards regression models

	Overall sample	Tertiles		
		1^a^	2	3
Individuals, *n* (%)	863 (100.0%)	287 (33.3%)	288 (33.4%)	288 (33.4%)
Deceased, *n* (%)	99 (11.5%)	26 (9.1%)	24 (8.3%)	49 (17.0%)
Plasma boron concentrations in µg/L	31.9 [22.9; 43.5]	20.5 [17.1; 22.9]	31.9 [28.7; 35.2]	50.9 [43.5; 60.5]

	HR [95% CI] for all-cause mortality associated with plasma boron			
	5-unit-increment			
Model 1	**1.07 [1.03–1.11]**	Ref	0.86 [0.49–1.50]	**1.85 [1.15–2.97]**
Model 2	1.03 [0.99–1.07]	Ref	0.57 [0.32–1.00]	1.01 [0.62–1.66]
Model 3	1.03 [0.99–1.07]	Ref	0.63 [0.35–1.12]	1.14 [0.68–1.93]

HR are provided for the second and third tertile as compared to the bottom tertile, and per 5-unit-increment in plasma boron. Associations are in hazard ratios [95% confidence interval] with bold figures indicating significant associations (*p* < 0.05). Model 1: unadjusted. Model 2: adjusted for sex and age. Model 3: Model 2 further adjusted for body mass index, waist-to-hip ratio, education, smoking status, and physical activity

*CI* confidence interval, *HR* hazard ratio

^a^Tertile 1 was set as reference

### Sex-stratified analyses

In sex-stratified analyses, higher plasma boron concentrations were significantly associated with poorer survival (HR: 1.11 [95% CI 1.03–1.18]) in women (27 deaths), but not in men (72 deaths, HR: 1.00 [95% CI 0.95–1.06], *p*_interaction_ = 0.034, Table 3).

**Table 3:** Sex-stratified association of plasma boron concentrations with all-cause mortality using Cox proportional hazards regression models

	HR [95% CI] for all-cause mortality associated with plasma boron		
	Men (*n* = 494)	Women (*n* = 369)	*p* value for interaction
Plasma boron concentrations as 5-unit-increment	1.00 [0.95–1.06]	**1.11 [1.03–1.18]**	**0.034**

Associations are in hazard quotient [95% confidence interval] with bold figures indicating statistical significance. Analysis was adjusted for age, body mass index, waist-to-hip ratio, education, smoking status, and physical activity

*CI* confidence interval, *HR* hazard ratio

### Restricted cubic splines

By use of restricted cubic splines regression, we tested for nonlinear associations between plasma boron concentrations and all-cause mortality. Neither in the overall sample (*p* = 0.424), nor in the male (*p* = 0.158) or female subsample (*p* = 0.425), a nonlinear association reached statistical significance.

### Exploratory dietary pattern by RRR and its association with survival

RRR in our analytical sample (*n* = 863) identified a dietary pattern that explained 31.2% of the variance in plasma boron concentrations and was characterized by high intake of fruits, nuts and seeds, and wine, as well as low intake of snacks, soft drinks, margarine, poultry, processed meat, and bread. The resulting dietary pattern was not associated with all-cause mortality when considered as 1-SD increment, neither in the overall sample (HR: 1.07 [95% CI 0.82–1.39], Table 4) nor when only considering women (HR: 0.98 [95% CI 0.40–2.67]).

**Table 4:** Association of an exploratory dietary pattern explaining maximal variation in ln-transformed plasma boron concentrations with all-cause mortality risk using Cox proportional hazards regression models

	Overall sample
Individuals, *n* (%)	863 (100.0%)
Deceased, *n* (%)	99 (11.5%)
Plasma boron concentrations in µg/L	31.9 [22.9; 43.5]

	HR [95% CI] for all-cause mortality associated with 1-SD increment in dietary pattern
Model 1	1.08 [0.85–1.37]
Model 2	0.92 [0.71–1.19]
Model 3	1.07 [0.82–1.39]

Associations are in hazard ratios [95% confidence interval]. Model 1: unadjusted. Model 2: adjusted for sex and age. Model 3: Model 2 further adjusted for body mass index, waist-to-hip ratio, education, smoking status, total energy intake, and physical activity

*CI* confidence interval, *HR* hazard ratio, *SD* standard deviation

## Discussion

In a moderate-sized population-based sample from northern Germany, plasma boron concentrations were associated with an increase in all-cause mortality risk in women, but not in men or the overall sample. However, the analyses in the female subsample were based on a relatively low number of deaths (*n* = 27). An RRR-derived exploratory dietary pattern explaining 31.2% of variation of plasma boron concentrations was also not associated with all-cause mortality.

The biological effects of boron remain controversial. In various animal models and human intervention studies, a high intake of boron was repeatedly associated with beneficial health outcomes, including immunostimulant effects and lower susceptibility to osteoporosis [1, 2]. Very high intake of boron, on the other hand, was found to be developmentally toxic in some studies in rodents during gestation. Yet, boron amounts administered in these studies well exceeded highest achieved boron exposures in humans and did not result in maternal mortality [5]. In a previous analysis conducted in a subsample of the popgen “control cohort” (similar to the one used in this manuscript), plasma boron was associated with a healthier diet, lower BMI, and lower levels of circulating C-reactive protein [3]. Contrasting the positive health effects of boron reported in the majority of previous studies, we observed that higher plasma boron concentrations conferred an increased risk of mortality in women over the course of 11 years. However, we cannot exclude that this is a chance finding, given the rather low number of deaths in women.

Data on mortality risk in relation to boron in humans are still sparse. In a cohort of *n* = 693 kidney transplant recipients, higher urinary boron excretion was associated with lower all-cause mortality, with no differences between men and women [6]. Likewise, in northern France, geographical areas with higher content of boron in drinking water showed a decreased mortality rate compared to areas with low boron levels in water [4]. This contrasts our findings of poorer survival associated with higher plasma boron concentrations in women, but not in men or the overall sample. The reasons for this sex difference in the association of boron with mortality are not clear. However, the reported interaction of boron with sexual hormones, e.g., by increasing levels of both testosterone and 17 beta estradiol after oral boron supplementation in postmenopausal women [9], might be a contributing factor. More studies are warranted that specifically target sex differences with regard to the association between boron and health outcomes.

In a small study on healthy women, a higher intake of boron-rich foods led to beneficial effects on lipid metabolism and body weight. However, this effect might have been conferred by a higher intake of other dietary compounds in boron-rich foods [10]. Accordingly, we aimed to assess if the higher mortality risk associated with higher plasma boron concentrations seen in women in our sample might be due to an underlying dietary pattern influencing plasma boron concentrations and, additionally, exerting detrimental effects on health. Even though the RRR-derived dietary pattern mirrored a boron-rich diet (high intake of fruits, nuts, and wine, low intake of animal products), greater adherence to this dietary pattern was not associated with survival, emphasizing the relevance of plasma boron itself in this context. Of note, as only the study sample sizes differed slightly between this study and the one previously reported by Weber et al. [3], this RRR-derived dietary pattern is very similar to the one previously published by our work group.

Noteworthy, most studies relating boron to beneficial effects on human health were interventional studies with dietary boron supplementation focusing on short- to middle-term effects. Contrastingly, in our prospective cohort, participants were followed for a median of 11 years, allowing for a long-term analysis of baseline plasma boron concentrations in relation to survival, thereby potentially accounting for a long-term impact of boron on health not captured in short-term intervention studies. We have to acknowledge, though, that plasma boron concentrations were only available at one point in time (the baseline examination). Therefore, we could not account for possible changes in plasma boron over time and cannot demonstrate with certainty that a single boron measurement is representative of the long-term boron status. Furthermore, we cannot exclude residual confounding through factors and variables not considered in our statistical models. In general, findings of this study have to be interpreted with caution, as the association between plasma boron concentrations and mortality risk in women might be a chance finding due to a small number of events in this subsample.

## Conclusion

In a population-based sample from northern Germany, we observed that higher plasma boron concentrations conferred an increased risk of all-cause mortality over 11 years in women, but neither in men nor in the overall sample. This is contrary to the previous reports on beneficial health effects associated with higher intakes of dietary boron or with higher levels of circulating boron in humans. These conflicting results combined with the limitation that results of this study are only based on a small number of deaths in women, highlight the need for future studies to further elucidate the relations between boron and human health, particularly with regard to possible sex differences and long-term effects.

## Data Availability

Data described in the article, code book, and analytic code will be made available upon reasonable request through our P2N data application platform (https://portal.popgen.de/).
